# The Effects of Ozone Sterilization on the Chemical and Mechanical Properties of 3D-Printed Biocompatible PMMA

**DOI:** 10.3390/mi15040472

**Published:** 2024-03-29

**Authors:** Ivan Morosavljević, Dražan Kozak, Tihomir Kosor, Janko Morosavljević, Luka Ferlič, Nenad Gubeljak

**Affiliations:** 1 Mechanical Engineering Faculty in Slavonski Brod, University of Slavonski Brod, 35000 Slavonski Brod, Croatia; dkozak@unisb.hr (D.K.); jmorosavljevic@unisb.hr (J.M.); 2 Faculty of Chemistry and Technology, University of Split, 21000 Split, Croatia; t.kosor@kalun.hr; 3 Faculty of Mechanical Engineering, University of Maribor, 2000 Maribor, Slovenia; luka.ferlic@um.si (L.F.); nenad.gubeljak@um.si (N.G.)

**Keywords:** ozone sterilization, poly(methyl-methacrylate) (PMMA), polymer oxidation, personalized medical device, environmentally friendly, fused deposition modeling (FDM)

## Abstract

Since ozone is highly corrosive, it can substantially affect the mechanical and chemical properties of the materials; consequently, it could affect the applicability of those materials in medical applications. The effect of ozone sterilization on the chemical and mechanical properties of additively manufactured specimens of biocompatible poly(methyl-methacrylate) was observed. FDM 3D-printed specimens of biocompatible PMMA in groups of five were exposed to high concentrations of ozone generated by corona discharge for different durations and at different ozone concentrations inside an enclosed chamber with embedded and calibrated ozone, temperature, and humidity sensors. A novel approach using laser-induced fluorescence (LIF) and spark-discharge optical emission spectrometry (SD-OES) was used to determine an eventual change in the chemical composition of specimens. Mechanical properties were determined by testing the tensile strength and Young’s modulus. A calibrated digital microscope was used to observe the eventual degradation of material on the surface of the specimens. SD-OES and LIF analysis results do not show any detectable sterilization-caused chemical degradation, and no substantial difference in mechanical properties was detected. There was no detectable surface degradation observed under the digital microscope. The results obtained suggest that ozone sterilization appears to be a suitable technique for sterilizing PMMA medical devices.

## 1. Introduction

Pathogenic bacteria and viruses in medical environments can lead to treatment complications, especially in surgical interventions, where nowadays additive-manufactured personalized medical devices are frequently used. Custom-made, precisely tailored implants generated by computer-assisted techniques and the patient’s individual computed tomography scans can nowadays be prefabricated to achieve better patient satisfaction, especially in craniofacial surgical interventions, compared to hand-shaped intraoperative implant molding [1].

Currently, the most commonly used alloplastic materials in surgical interventions besides autologous bone grafts are PMMA, hydroxyapatite, and titanium, although in recent studies and clinical trials, other materials such as PEEK, porous polyethylene, polyethylene terephthalate glycol (PETG), and carbon fibers have been used [1]. PMMA is the most extensively used biocompatible material in cranioplasty and dentistry because it is well tolerated by tissue, easily placed and modified, light in weight, inexpensive, radiolucent, non-conductive, and radiates less heat compared to metal materials [1,2].

According to Münker et al. [3], ethylene oxide, hydrogen peroxide gas plasma, and γ-irradiation appear to be suitable techniques to sterilize PMMA personalized medical devices, and autoclave sterilization is not suitable because it causes deformations and exfoliation during the sterilization process. According to Zeng et al. [4], ethylene oxide can cause numerous cardiovascular diseases and poses a threat even to the residents near the sterilization facility through secondhand inhalation, and it is a known human carcinogen according to the International Agency for Research on Cancer (IARC) [5]. Both hydrogen peroxide and γ-irradiation sterilization processes generate hazardous waste and require expensive and specialized equipment. Moreover, γ-irradiation sterilization processes cause a decrease in the molecular weight of PMMA due to chain scission, which directly correlates to the worsening of the mechanical properties [3].

Ozone sterilization is an environmentally friendly and cost-effective method to achieve material disinfection. The efficiency of ozone’s oxidative potential on common microorganisms has been extensively studied, particularly in the food and water treatment industries. The effect of ozone sterilization on most biocompatible alloplastic materials is widely unknown, and since ozone is highly corrosive, it can substantially affect the mechanical and chemical properties of the materials and, consequently, the applicability of those materials in medical applications. The effect of ozone sterilization on the chemical properties, surface morphology, and mechanical properties of PMMA is unknown; therefore, this study aims to investigate the effect of ozone on tensile properties and changes in the chemical composition of PMMA.

According to Epelle et al. [6], the sufficient ozone concentration and exposure time that yield complete microbial removal for *A. fumigatus*, *C. albicans*, *E. coli*, and *S. aureus* are 20 ppm and 4 min at 20 °C and 50% relative humidity, respectively. Another study by Sharma et al. [7] suggests that an ozone dosage of 25 ppm at 90% RH for 20 min is sufficient to inactivate 15 different species of medically important bacteria. In the case of the virucidal properties of ozone, a few studies are available, and these were carried out using very different experimental methods for the assessment of ozone virucidal efficacy. Amidst recent SARS-CoV-2 pandemics, many studies [8,9] were performed on the potential of ozone for disinfection properties in large spaces (hospitals) and personal protective equipment. Among these, Yano et al. [8] reported the first in vitro inactivation study on SARS-CoV-2 by only using 6 ppm ozone at room temperature, obtaining a 3.3log_10_ (99.99%) reduction from 0.1 to 0.4 mgL^−1^ in only 55 min. Bayarri et al. [9] reported that ozone can be an effective disinfectant in the gas phase, successfully inactivating up to 28 different viruses of the 29 tested with ozone concentrations under 20 ppm in only 70 min, except Bacteriophage MS2, which requires higher concentrations.

Ozone is a highly thermodynamically unstable triatomic molecule containing three oxygen atoms, which, depending on ambient conditions like temperature and pressure, decompose to pure oxygen with a half-life of 40 min at 20 °C and about 140 min at 0 °C [10]. Because of the short half-life at the ground temperature and pressure, ozone decomposes back to diatomic oxygen, leaving no toxic residue or emission of harmful pollutants. The most widely used processes to produce ozone are based on the following reaction [11]:

(1) $${3O}_{2}+68.4\;Kcal\to 2O_{3}$$

Two technologies are most frequently used to produce ozone: ultraviolet and corona discharge ozone generators. When diatomic oxygen molecules are exposed to high ultraviolet radiation, ozone is generated; therefore, UV ozone generators fundamentally mimic the natural process that’s occurring in the stratosphere. Corona discharge ozone generators produce ozone when the flow of air or pure diatomic oxygen passes through a high-voltage field generated between two electrodes separated by dielectric material, which is coated or bonded to one of the electrodes. Corona discharge forms in the air gap between the dielectric surface and electrode when sufficient voltage is applied between the two electrodes. The voltage required for corona discharge to occur depends on the gap between the electrode and dielectric surface and the characteristics of the dielectric material. Diatomic oxygen molecules break on atomic oxygen, which bonds with other diatomic oxygen molecules passing through an electric field and forming ozone [12].

Since ozone can be produced with portable equipment without any hazardous waste and with a relatively short half-life, it could be potentially utilized in a fused deposition modeling 3D printing process to achieve a sterile personalized medical device in a single manufacturing step by equipping a 3D printer chamber with an ozone circulation source, for example, from a tube corona discharge ozone generator, and a regulation system to maintain a desired ozone concentration.

## 2. Materials and Methods

A total of 20 specimens were made of biocompatible medical-grade PMMA filament by Novus Life Sciences Ltd., Shatin, NT, Hong Kong, China, using fused-deposition modeling (FDM) 3D printing technology with printing parameters described in Table 1. To determine the tensile properties, all specimens were made according to the ISO 527-2 standard, type 5A [13]. Specimens were printed using an ENDER 3 PRO FDM 3D printer at room temperature and humidity. The 3D model was made using Autodesk Fusion v.2.0.16985 software, and toolpaths were generated using Ultimaker CURA 4.13.1. software.

### 2.1. Sterilization Process

**Figure 1:**
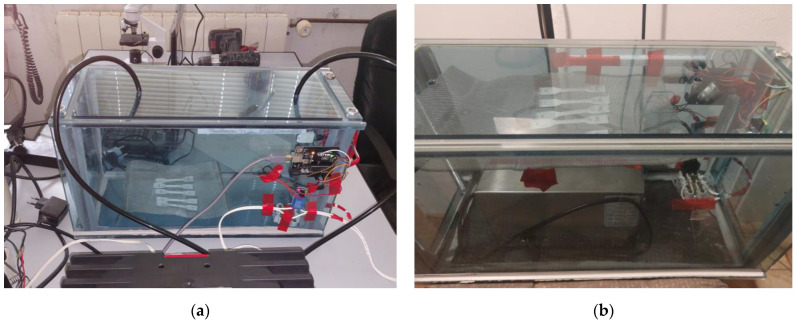
(**a**) 5 g/h ozonation chamber setup, and (**b**) 60 g/h ozonation chamber setup.

Sterilization was carried out in an enclosed 12 mm thick PMMA chamber by using a 5 g/h tube corona discharge ozone generator and a diaphragm air pump with a 4 L/min capacity for SG1 and SG2, respectively, as shown in Figure 1a. The chamber was connected to the pump suction and pressure sides to achieve a closed-loop system. To achieve higher concentrations, SG3 sterilization was carried out by using a Vosoco 100 W 60 g/h corona discharge ozone generator inside a chamber, as shown in Figure 1b. In both cases, specimens were placed on 500 µm EN 10088-3:2005 1.4404 [14] (AISI 316L) stainless steel mesh. The chamber was equipped with an Adafruit DHT22 temperature and humidity sensor manufactured by Asair, sourced from TouZhanTeng HK ltd, Kowloon, Hong Kong, and a calibrated Winsen, Zhengzhou, China, MQ131, high-concentration ozone gas concentration sensor. For regulation and data reading, a Lafin uno R3 board, Shenzhen Hong Shu Yuan Technology ltd., Shenzhen, Guangdong, China equipped with an ATmega328P microcontroller, and a relay switch was used to ensure PID regulation of ozone concentration.

**Figure 2:**
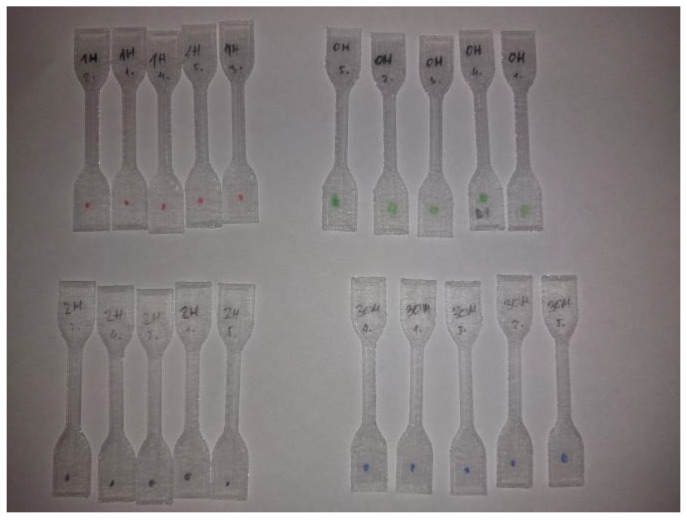
FDM 3D-printed PMMA specimens with test designations.

As shown in Figure 2, specimens were divided into 4 groups of 5, where specimen group 0 (SG0) represents an unsterilized control group and groups 1 (SG1), 2 (SG2), and 3 (SG3) are sterilized groups with different sterilization durations and concentrations.

**Figure 3:**
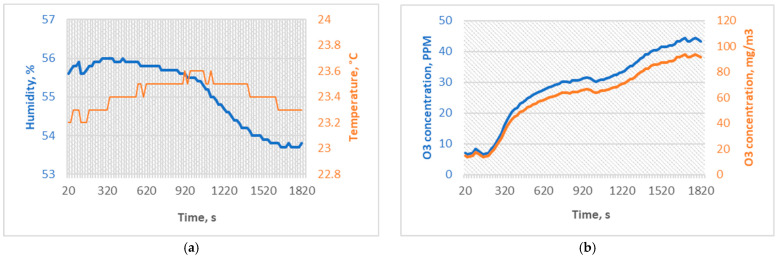
(**a**) Temperature and humidity sterilization conditions for SG1, and (**b**) ozone concentrations of the sterilization process for SG1.

Since this experiment aims to investigate whether the ozone is causing a chemical degradation in PMMA or a change in the mechanical properties of the material, experimental data were recorded from the moment when ozone was introduced into the chamber rather than when a certain concentration was reached. Temperature and humidity sensors were programmed to work in continuous mode because a temperature fail-safe loop, ozone concentration sensor, and data reading interval were set to 20 s. SG1 was sterilized for 30 min with an average ozone concentration of 28.88 ppm (61.04 mg/m^3^) and a 45 ppm peak concentration. Regulation circuitry was programmed to activate a relay switch on a 45 ppm concentration of ozone, and a safety switch was programmed to break the power source to the ozone generator if 40 °C is read by the sensor within a chamber. SG1 experiment conditions were determined similar to the Sharma et al. [7] study, where 25 ppm of ozone dosage for 20 min was used, with a time compensation of ten minutes to reach the concentration in the chamber. The SG1 experiment conditions are shown in Figure 3.

**Figure 4:**
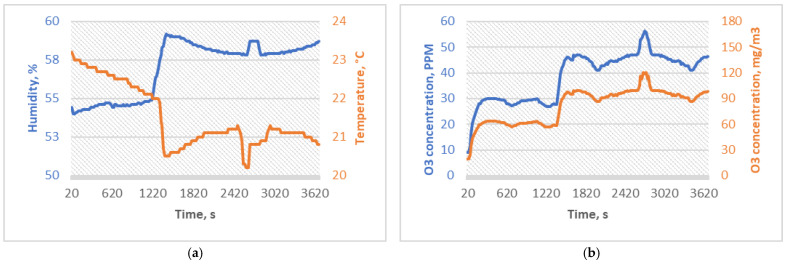
(**a**) Temperature and humidity sterilization conditions for SG2, and (**b**) ozone concentrations of the sterilization process for SG2.

SG2 experiment conditions were determined similarly to those of Yano et al. [8] and Bayarri et al. [9] studies, which reported the deactivation of most medically relevant viruses within 60 and 70 min periods with ozone concentrations up to 20 ppm; therefore, the SG2 group was sterilized for 60 min with an average of 38.62 ppm (81.71 mg/m^3^) and a peak ozone concentration of 59 ppm. Another goal of this experimental group was to observe whether increased concentrations induce a more significant change in the mechanical and chemical properties of PMMA specimens; the average ozone concentration was increased compared to SG1 by 30%. The SG2 experiment conditions are shown in Figure 4.

**Figure 5:**
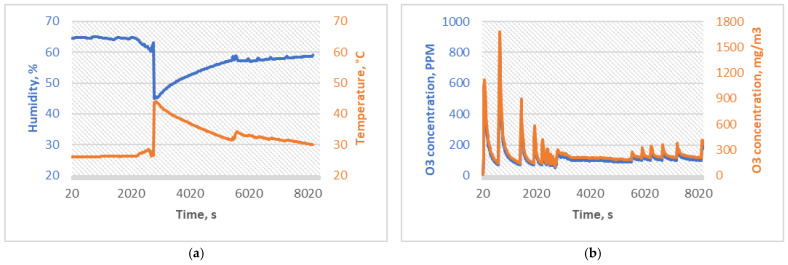
(**a**) Temperature and humidity sterilization conditions for SG3, and (**b**) ozone concentrations of the sterilization process for SG3.

The SG3 experiment was designed to increase the ozone concentration to the highest safely attainable ozone concentration with available equipment. The sterilization duration was doubled compared to the SG2, and the ozone concentration average was 121.18 ppm (256.12 mg/m^3^), with a peak value of 795 ppm. Since a powerful 100 W ozone generator was used inside a sterilization chamber, temperature was a limitation of this experiment to achieve even higher concentrations. PID regulation was programmed into a microcontroller with a set 40 °C temperature and a 180 ppm ozone concentration limit switch. The time between the ozone sensor and data readings was set to 20 s. To achieve a homogenous mixture of air and ozone inside a chamber and to avoid the influence of density differences between air and ozone, a 100 mm radial fan with a 0.6 m^3^/h flow rate was used at an outlet of the corona discharge ozone generator. SG3 experimental conditions are shown in Figure 5.

### 2.2. Spark-Discharge Optical Emission Spectroscopy (SD-OES)

As reported in the latest studies, plasma-forming analysis methods are an emerging tool for the detection of polymer degradation [15,16]. Recent studies [17] show a high correlation between analysis data of polymer degradation in a corrosive atmosphere obtained by FTIR analysis and that obtained using the C_2_ Swan band from plasma forming methods, such as laser-induced breakdown spectroscopy (LIBS). The C_2_ band-forming mechanism is explained in other studies [18]. Therefore, in this study, the SD-OES system is used to detect eventual changes in the C_2_ Swan molecular band to evaluate potential material degradation caused by sterilization. Spark discharge is created by a high-voltage impulse generator operating at 20,000 V and 10 Hz frequency in the air atmosphere over a 28 mm^2^ surface area of the samples between DIN EN 10088-3 1.4571 [14] (AISI 316Ti) electrodes. Emission spectra are obtained using the ASEQ instrument’s LR1 UV-VIS spectrometer, Vancouver, Canada. Spectra are averaged over 1000 ms intervals and normalized to the highest peak value.

**Figure 6:**
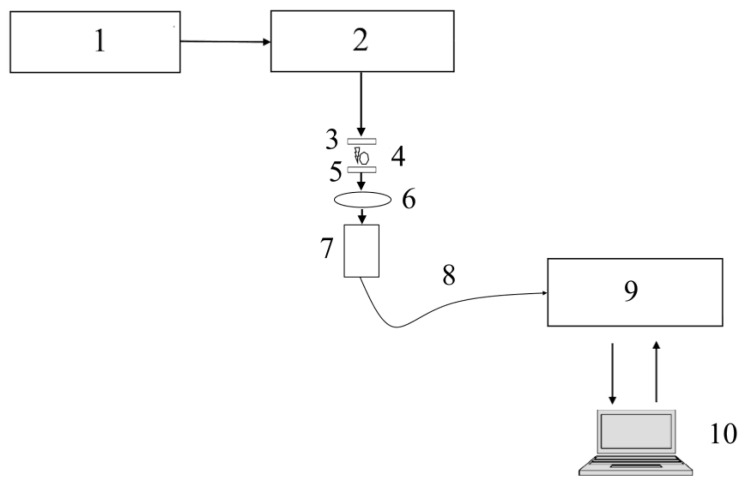
A schematic representation of the SD-OES system. The components of the system include: (1) high voltage source, (2) pulse generator, (3) high-voltage anode, (4) sample, (5) high-voltage cathode, (6) gathering lens, (7) light gathering optics, (8) optical fiber, (9) spectrometer, and (10) data acquisition and control.

The SD-OES principal system is shown in Figure 6. The operating voltage of 20,000 V is often generated by the high-voltage source, such as the combination of a transformer and full-bridge rectifier or a full-bridge rectifier paired with a Villard cascade. A pulse generator circuit is used to generate precisely timed sparks between the cathode and anode, which is an important parameter for the data acquisition and control unit. The spark generated between the electrodes combusts the sample instantly, creating plasma. Gathering optics gather plasma spectra through a lens that is transferred to a spectrometer through an optical fiber cable. The acquisition and control unit reads and interprets information received from a spectrometer and controls the next pulse generated by the pulse generator. The SD-OES system operates similarly to LIBS; the difference is a source generating plasma from the sample, whereas in SD-OES, an electric arc between electrodes is used instead of a laser pulse paired with focusing lenses.

### 2.3. Laser-Induced Fluorescence (LIF)

Laser-induced fluorescence (LIF) was used to evaluate possible structural and chemical changes in the sample. LIF was arranged in a transmission setup using 2 mm thick PMMA samples. Fluorescence excitation was obtained by a 50 mW, 451 nm diode laser. UV-Vis absorption spectra were determined using an LR1 ASEQ instrument UV-VIS spectrometer, Vancouver, Canada. To prevent saturation of the detector by Rayleigh scattering, a 460 nm cut-off long-pass filter was used. Spectra were obtained at 2000 ms exposure, and 64 scans per sample were averaged. Since the fluorescence response from PMMA is unlikely to be from the bulk molecules, impurities and defects in the material are expected to play a significant role, and any degradation in the material should induce detectable changes in the fluorescence spectra of the samples [19,20,21].

**Figure 7:**
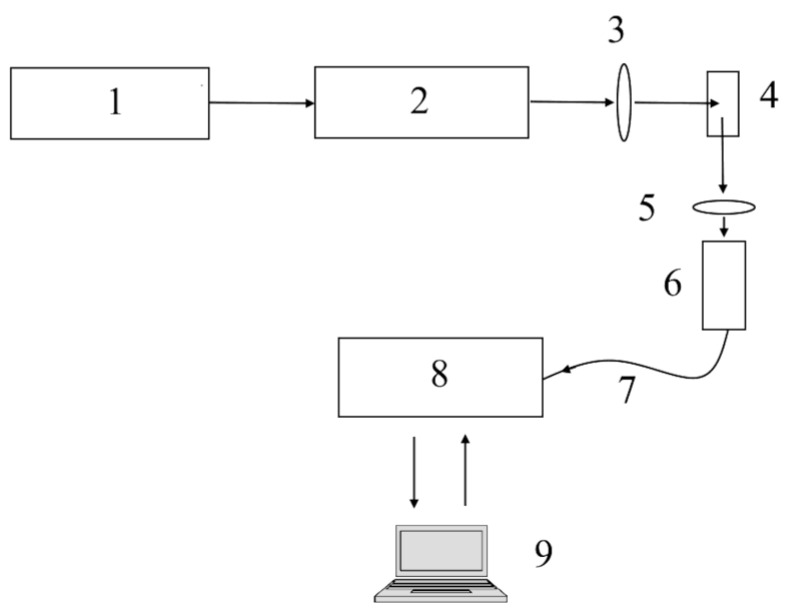
A schematic representation of the LIFs system. The components of the system include: (1) pulse width modulator module, (2) continuous wave laser, (3) lens, (4) sample, (5) laser blocking lens, (6) light gathering optics, (7) optical fiber, (8) spectrometer, and (9) data acquisition and control.

A schematic of the LIFs system is shown in Figure 7. A 50 mW, 451 nm diode laser is powered by a power width modulation module, which controls the average power emitted by the laser. A laser beam is focused on the sample using a lens, and a spectrometer reads the optical signal through light-gathering optics and a cut-off long-pass filter to prevent a Raylight scattering effect. The acquisition and control unit controls the PWM module and reads data acquired from the spectrometer.

### 2.4. Surface Morphology

The AmScope MU120-FL microscope digital camera with a Sony Exmor CMOS sensor, AmScope, Irvine, USA, was used for visual detection of eventual morphological changes on the surface of the specimens. The surfaces of the specimens were observed under 40× and 10× magnification, respectively.

### 2.5. Tensile Strength

**Figure 8:**
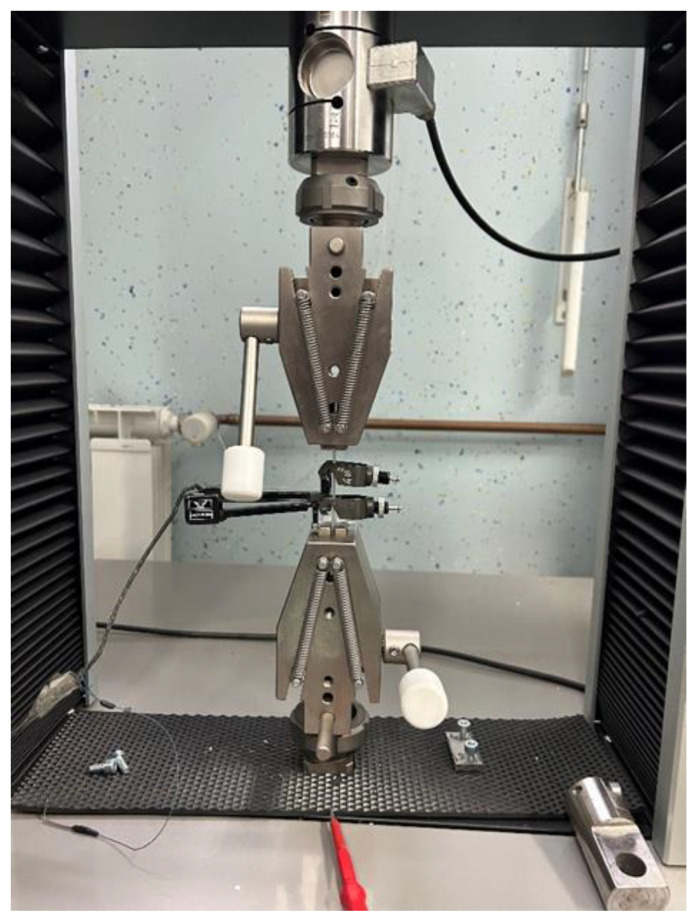
Setup for testing with proportional grips and an extensometer.

A total of four specimens from each SG were used to obtain tensile properties and to detect ozone-influenced changes in material properties. The fused deposition modeling technique, also known as fused filament fabrication, is an additive manufacturing process within the realm of material extrusion in the 3D printing process; therefore, it builds parts layer by layer by selectively extruding material through a programmed path, leaving a rough surface finish caused by seams between extruder layers. To avoid the influence of roughness on the obtained tensile results, each specimen was sanded and water-polished from both sides; thereafter, the nominal cross-section was bright, and the thickness of all specimens was 4.5 and 1.2 mm, respectively. Tensile strength was measured in the Galdabini Quasar 5 testing machine, Galdabini, Cardano al Campo, Italy, according to ISO 527-1 [22] and ISO 527-2 standards. A loading cell with a maximum capacity of 500 N and +0.5% accuracy was used. Elongation was measured by an INSTRON extensometer, Labsys d.o.o., Ljubljana, Slovenia, with a 12.5 mm initial length and +/−5 mm displacement with 0.1% accuracy. Tests were performed in stroke control with constant 1mm/min velocity at 23.5 °C room temperature and 48% humidity. The setup for testing with proportional grips is shown in Figure 8. During the test, a force-extension plot was recorded for each specimen.

## 3. Results

### 3.1. Surface Morphology

**Figure 9:**
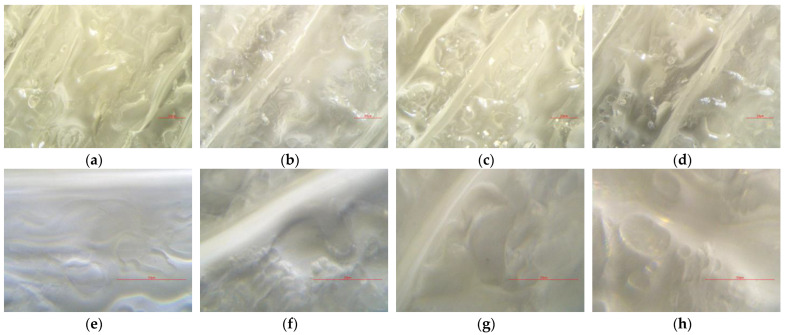
Specimens surface under 10× and 40× magnification: (**a**) SG0 under 10× magnification, (**b**) SG1 under 10× magnification, (**c**) SG2 under 10× magnification, (**d**) SG3 under 10× magnification, (**e**) SG0 under 40× magnification, (**f**) SG1 under 40× magnification, (**g**) SG2 under 40× magnification, and (**h**) SG3 under 40× magnification.

The effect of sterilization on surface degradation was evaluated using a calibrated digital microscope on untreated and treated PMMA specimens, as shown in Figure 9. Extruder layer seams were deliberately targeted under a microscope to evaluate whether ozone caused fractures or cavities on the seam of extruder passes. Comparing the results of the microscope scans, there are no visible crack formations or seam corrosion formations on the surface of the specimens on either of the scans. All four specimens have visible trapped air bubbles under their surfaces; therefore, further research is required to investigate the FDM 3D printing parameters, specimen cooling, and environmental conditions that could potentially affect the formation of the air bubbles in PMMA during the extruding process.

### 3.2. SD-OES

**Figure 10:**
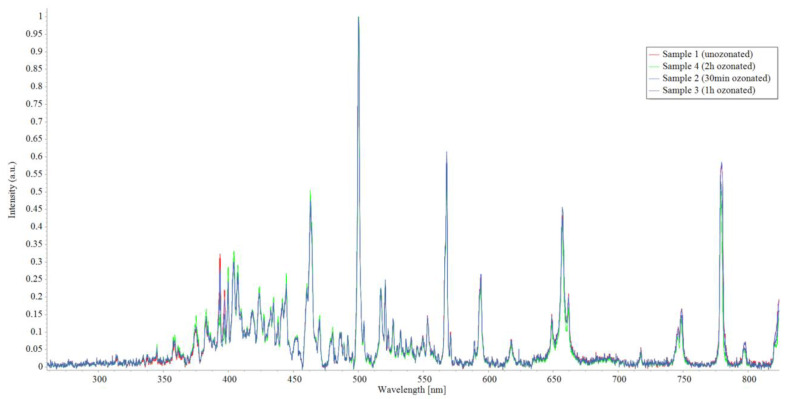
Optical emission spectra generated by spark discharge of all SG samples.

**Figure 11:**
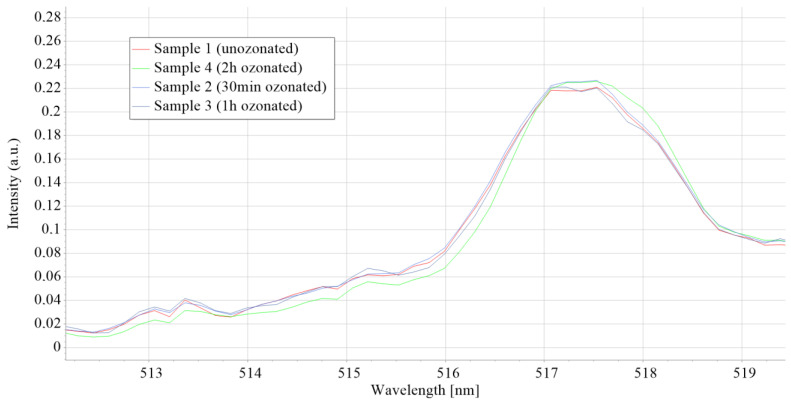
Optical emission spectra generated by spark discharge of all SG samples.

The optical emission spectra shown in Figure 10 represent emission spectra averaging over 1000 ms intervals and normalized on the highest peak value of all 4 samples of the unsterilized and sterilized materials during different time intervals. In optical emission spectrometry, the atomic emission of a specific element is usually connected with a single line at a specific wavelength, and molecular bands relate to the vibrational and rotational states of ablated diatomic species, for example, the C_2_ molecule. In plasma-forming optical-emission spectrometry methods, the bands (C_2_ molecules), also called carbon dimers, are usually shown in the range of 465–590 nm. The Swan band is located at a wavelength of 516.5 nm [17], and there is a high correlation between the analysis data of polymer degradation in a corrosive atmosphere obtained by FTIR analysis and that obtained using the C_2_ Swan band from plasma forming methods such as LIBS. The C_2_ Swan bands of all SGs are shown in Figure 11.

**Figure 12:**
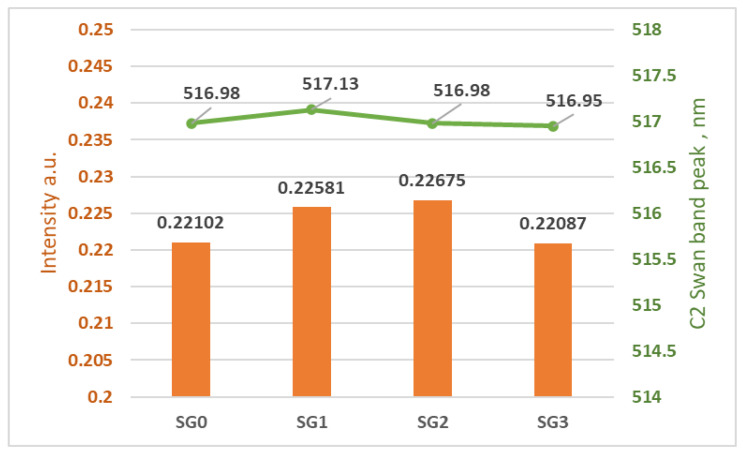
C2 Swan band data for each SG.

According to the C_2_ Swan band results shown in Figure 11, there are no visible changes in the optical emission spectra at a characteristic C_2_ Swan band wavelength nor a clear correlation between ozone concentrations and sterilization durations with the change in the C_2_ Swan band intensity; the results suggest that there is no detectable ozone sterilization degradation of the material. Precise data on the peaks shown in Figure 11 is shown in Figure 12.

### 3.3. Laser-Induced Fluorescence

**Figure 13:**
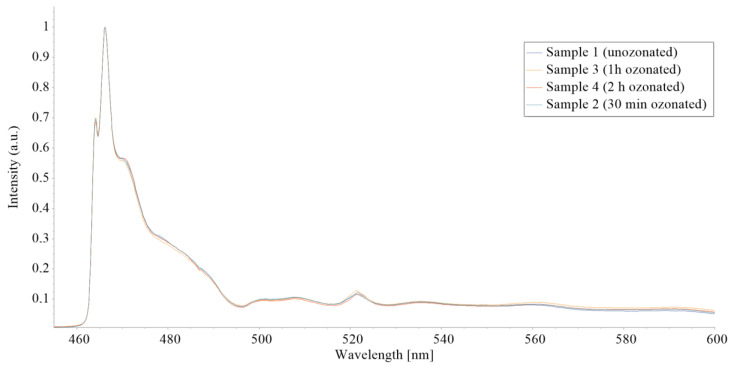
Laser-induced fluorescence of all four sample groups.

All the samples show the highest fluorescence band at 466 nm, with characteristic shoulders at 464 and 470 nm. Also, all the samples show a band at 521.5 nm that corresponds to Stokes light scattering assigned to C–H and O–CH3 band vibrations [23,24]. As shown in Figure 13, there are no detectable changes in the LIF spectrum of the samples that would suggest ozone-caused degradation.

### 3.4. Mechanical Properties

**Figure 14:**
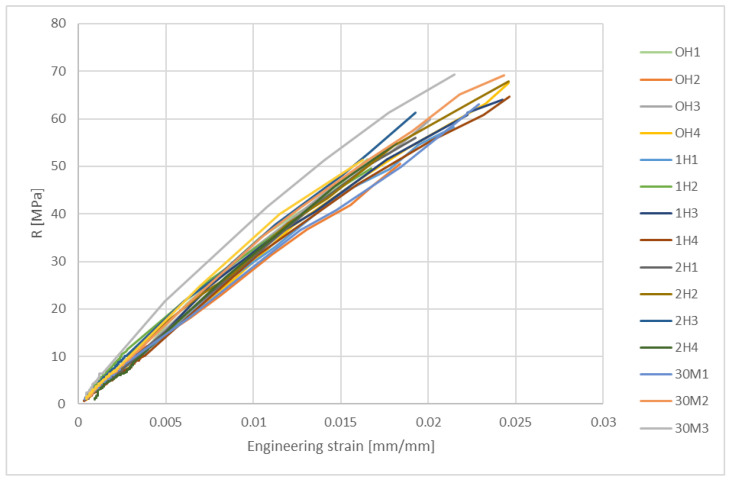
Stress–strain curves for each SG with 4 samples per group.

Mechanical properties are obtained and analyzed according to standards ISO 527-1 [22] and ISO 527-2 [13]. Results for all 16 specimens are shown in Figure 14 by taking into account the cross-section of each tested specimen after the polishing process.

**Figure 15:**
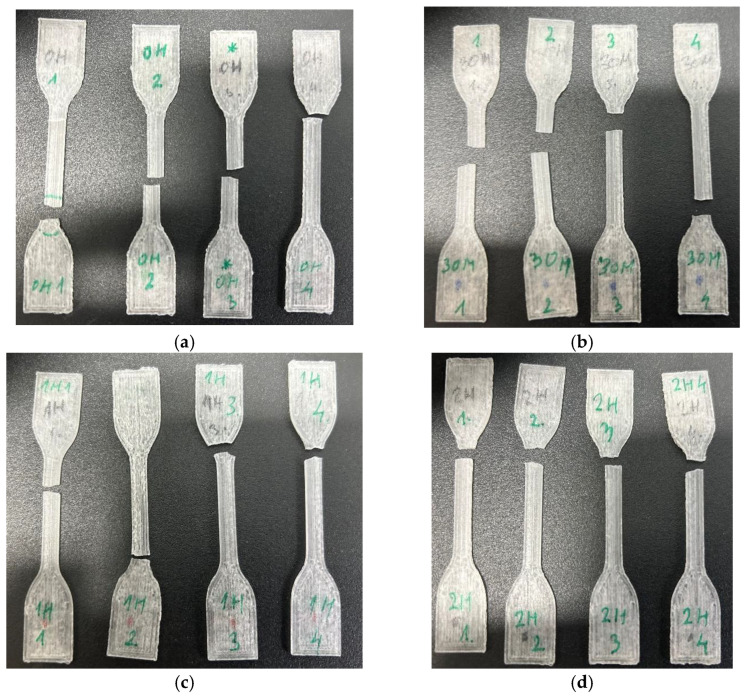
(**a**) Tensile test failure points of SG0, (**b**) tensile test failure points of SG1, (**c**) tensile test failure points of SG2, and (**d**) tensile test failure points of SG3.

Figure 14 results suggest that all specimens exhibit brittle material properties, breaking without yielding at low strain. Table 2 shows the averaged values of Young’s modulus, tensile strength, and elongation, according to the ISO 527-1 standard [22] for each SG, respectively. Furthermore, the 0H3 specimen in Figure 14 shows a steeper stress–strain curve compared to the other specimens. All of the specimens were stored in an air-tight container separately after the ozone sterilization, and a higher Young modulus of a 0H3 (designated with a “*” mark under Figure 15) specimen could indicate container failure according to Radojičić et al. [25] or an oscillation in 3D printing bed temperature according to Abdel-Wahab et al. [26].

The values listed in Table 2 correspond to those listed in the literature, where the modulus of elasticity E is between 2.8 and 3.2 GPa and the tensile strength is between 60 and 70 MPa for acetals and acrylic polymers [27]. The failure points of the specimens are shown in Figure 15.

## 4. Discussion

The purpose of this study was to investigate the influence of ozone sterilization on the mechanical and chemical properties of biocompatible 3D-printed PMMA personalized medical devices. Namely, sterilization procedures on 3D-printed personalized medical devices made of PMMA are predominantly used for cranial surgical interventions and dental procedures. Recently, prefabrication of additively manufactured customized implants has been introduced in clinical trials and standard procedures to overcome the shortcomings of intraoperative molding and enable surgeons to achieve better results in the restoration of the preoperative contours, therefore improving aesthetic outcomes and decreasing operating time and the risks of blood loss and infection. Other sterilization methods on PMMA biocompatible personalized medical devices described in a recent study by Münker et al. [3], such as ethylene oxide, hydrogen peroxide gas plasma, and γ-irradiation, appear to be suitable, but all of the above methods require specialized robust equipment, pose a handling threat to users and some even to sterilization facilities nearby residents, and leave toxic or carcinogenic waste, namely because those methods can hardly, if even possibly, be utilized within another process such as 3D printing. Ozone sterilization equipment is portable and inexpensive, and considering a short half-life of 40 min at ground pressure and temperature, ozone breakdown byproducts, and the required quantity if utilized within a 3D printing chamber, ozone poses a low threat to the users and the environment without any generated waste.

SD-OES and LIF results suggest that there are no detectable ozone-caused oxidative or corrosion effects on either of the SGs regarding different sterilization durations and concentrations.

**Figure 16:**
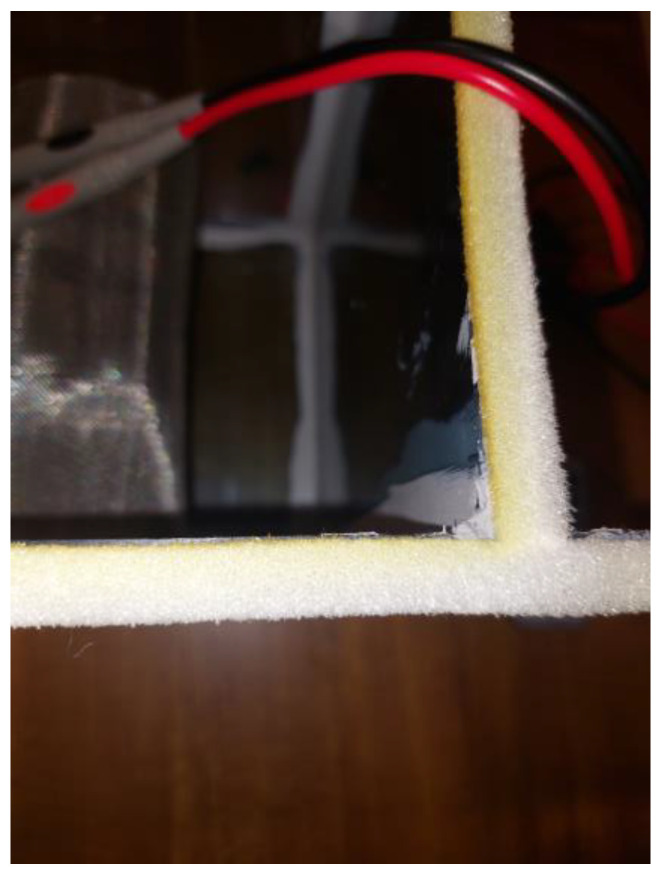
Ozone-caused corrosion of the polyurethane foam sealing strip on the sterilization chamber.

Furthermore, there is no detectable visible ozone-caused surface degradation of material under the digital microscope under 10× and 40× magnification, respectively. Visually, neither of the SGs changed morphology nor color compared to the PE seal on the test chamber, as shown in Figure 16. However, further study is required to investigate a correlation between printing parameters or environmental conditions and bubble formation in FDM 3D-printed specimens of PMMA.

No difference in obtained tensile test results was found between the specimen groups with varying ozonation durations and concentrations, and there is no clear correlation between ozonation duration and change in tensile strength or Young’s modulus, but further study is required to investigate the effect on other mechanical properties such as impact strength, hardness, and fracture toughness. All of the specimens demonstrate similar stress–strain curves shown in Figure 14 and Table 2, which correspond to available material data in the literature.

Further study is required to investigate the effect of ozone sterilization on elevated temperatures, for example, during a PMMA FDM 3D printing process to potentially integrate ozone sterilization into the additive manufacturing process, which could potentially produce sterile prints in a single manufacturing step.

## 5. Conclusions

This study provides an overview of the influences of ozone sterilization on the mechanical and chemical properties of biocompatible FDM 3D-printed, PMMA personalized medical devices. Ozone sterilization appears to be a suitable technique to sterilize personalized medical devices made of PMMA. Further study is required to investigate the effect of ozone on PMMA at elevated temperatures, for example, during an FDM 3D printing process, to potentially incorporate ozone sterilization within a 3D process.

## Figures and Tables

**Table 1:** 3D printing parameters.

Parameter	Unit	Value
Bed temperature	°C	90
Print speed	mm/s	50
Nozzle diameter	mm	0.4
Nozzle temperature	°C	245
Layer height	mm	0.2
Fill pattern	-	Lines
Print direction	-	Horizontal
Infill	%	100
Wall thickness	mm	0.8
Filament diameter	mm	1.75
Wall line count	-	3

**Table 2:** Average values of Young’s modulus, tensile strength, and elongation.

Specimen Group	SG0	SG1	SG2	SG3
E (GPa)	2.97	3.69	3.17	3.09
σ_T11_ (Mpa)	57.33	63.07	59.08	59.90
A (%)	1.91	2.18	2.22	1.97

## Data Availability

All experimental raw data in graphical form is included in the manuscript, and tensile data sets are available on demand from authors.
